# Shear Bond Strength of an Etch-and-rinse Adhesive to Er:YAG Laser- and/or Phosphoric Acid-treated Dentin

**DOI:** 10.5681/joddd.2013.012

**Published:** 2013-05-30

**Authors:** Abdolrahim Davari, Mostafa Sadeghi, Hamid Bakhshi

**Affiliations:** ^1^Associate Professor, Department of Restorative Dentistry, Faculty of Dentistry, Shahid Sadoughi University of Medical Sciences, Yazd, Iran; ^2^Professor, Department of Restorative Dentistry, Faculty of Dentistry, Rafsanjan University of Medical Sciences, Rafsanjan, Iran; ^3^Instructor, Medical Education Development Center, Rafsanjan University of Medical Sciences, Rafsanjan, Iran

**Keywords:** Acid etching, adhesion, Er:YAG laser, etch-and-rinse adhesive, shear bond strength

## Abstract

***Background and aims.*** Er:YAG laser irradiation has been claimed to improve the adhesive properties of dentin; therefore, it has been proposed as an alternative to acid etching. The aim of this in vitro study was to investigate the shear bond strength of an etch-and-rinse adhesive system to dentin surfaces following Er:YAG laser and/or phosphoric acid etching.

***Materials and methods.*** The roots of 75 sound maxillary premolars were sectioned below the CEJ and the crowns were embedded in auto-polymerizing acrylic resin with the buccal surfaces facing up. The buccal surfaces were ground using a diamond bur and polished until the dentin was exposed; the samples were randomly divided into five groups (n=15) according to the surface treatment: (1) acid etching; (2) laser etching; (3) laser etching followed by acid etching; (4) acid etching followed by laser etching and (5) no acid etching and no laser etching (control group). Composite resin rods (Point 4, Kerr Co) were bonded to treated dentin surfaces with an etch-and-rise adhesive system (Optibond FL, Kerr Co) and light-cured.After storage for two weeks at 37°C and 100% humidity and then thermocycling, bond strength was measured with a Zwick Universal Testing Machine at a crosshead speed of 1 mm/min. Data was analyzed using parametric and non-parametric tests (P<0.05).

***Results.*** Mean shear bond strength for acid etching (20.1±1.8 MPa) and acid+laser (15.6±3.5 MPa) groups were significantly higher than those for laser+acid (15.6±3.5 MPa), laser etching (14.1±3.4 MPa) and control (8.1±2.1 MPa) groups. However, there were no significant differences between acid etching and acid+laser groups, and between laser+acid and laser groups.

***Conclusion.*** When the cavity is prepared by bur, it is not necessary to etch the dentin surface by Er:YAG laser following acid etching and acid etching after laser etching.

## Introduction

The treatment of dental tissues prior to adhesive restorative procedures is an extremely important step in the bonding protocol and determines the clinical success of restorations. After the introduction of acid-etching technique for the pretreatment of dental hard tissues, adhesive materials and new techniques were originally developed to act on the tooth substrate prepared by conventional techniques.^1,5^ Nevertheless, a disadvantage attributed to acid etching is the demineralization of tooth structures, making them more permeable and prone to acid attacks, especially if the demineralized substrates are not completely filled by adhesive resins.^6^ In order to overcome this limitation, new investigations point to alternative techniques that could produce better effects than acids produce. Among these innovations for dentinal surface treatment, the use of lasers has been widely advocated.^7-9^

Dentin substrate is a complex structure which can influence the bonding of restorative systems; therefore, bonding to dentin surface is a greater challenge than to enamel surface. Conventional dentin bonding schemes also depend on an acid etchant to remove the smear layer and partially demineralize the dentin surface, expose collagen fibers, and widen the tubule lumen.^1,10^ The application of adhesive systems to etched dentin results in the formation of resin tags and a hybrid layer in which the demineralized collagen matrix is infiltrated by resin monomers that polymerize in situ, producing a higher bond strength.^11,12^

The use of low-energy lasers has increased for pretreatment of enamel and dentin in an attempt to optimize dental hard tissue conditioning in place of conventional acid etching for adhesive procedures.^1,13,14^ Laser irradiation on dental hard tissues is a process of continuous vaporization and micro-explosions resulting from vaporization of water trapped in the hydroxyapatite matrix.^10^ The effect of the laser on the dentin surface depends on various parameters such as the laser wavelength, pulse duration, the emission mode, energy density, frequency, tissue water content, air/water spray cooling, and the nature of any post-irradiation surface treatment such as acid etching, ultrasonic cleaning, or air abrasion.^6,15^

The Erbium:Yttrium–Aluminum–Garnet (Er:YAG) laser is a good candidate for safe and effective treatment of dentin surface, removing the smear layer, similar to acid etching, opening dentinal tubules and creating a microscopically rough surface with a micromechanical retention pattern, which is apparently ideal for adhesion.^5,6,11,13^ Moreover, cavity pretreatment with Er:YAG laser has been proposed as an alternative to acid etching of enamel and dentin.^13^ The Er:YAG laser emits a wavelength (2.94 μm) coincident with the main absorption band of water (3.0 μm), and it is also well absorbed in hydroxyapatite.^6,16,17^ There is some controversy concerning application of acid etching or laser irradiation for pretreatment of tooth surfaces.^1,6-8,18^ Some studies have shown no statistical difference in bond strength values between laser-irradiated and acid-etched dentin, suggesting that laser irradiation might replace acid etching as a pretreatment procedure for dentin bonding.^1,5,10,17,19^

Since a smear layer is often formed during cavity preparation, its removal has been considered important for obtaining good adhesion to dentin. Acid etching has been shown to be an efficient strategy for smear layer removal, exposing open dentinal tubules and a thin superficial layer of demineralized intertubular dentin.^12^ Therefore, several studies have demonstrated that phosphoric acid etching is better than laser irradiation for composite resin adhesion.^7-9,16,18^ Nevertheless, some studies have shown that bond strength values increase when the two procedures are combined.^1,9,20,21^

Considering the fact that Er:YAG laser surface treatment is a promising alternative for the conditioning of dentin surface, the bond strength to Er:YAG-lased tooth substrate reported in the literature is often confusing and even contradictory.^1,6-8,18^ Accordingly, this in vitro study was conducted to investigate the shear bond strength of a composite resin bonded with etch-and-rinse adhesive system to dentin treated with Er:YAG laser and/or phosphoric acid. The null hypothesis to be tested was that there was no difference in the shear bond strength between composite resin and dentin treated by laser and phosphoric acid using an etch-and-rinse adhesive system. The results of this study will determine whether it is possible to eliminate the acid-etching step of an etch-and-rinse adhesive by application of Er:YAG laser and whether it can improve the adhesion to dentin surfaces.

## Materials and Methods

The study protocol was approved by the Research Ethics Committee of Rafsanjan University of Medical Sciences, Iran. Seventy-five sound human maxillary premolars, which had freshly been extracted for orthodontic reasons were used for the purpose of this in vitro study. After extraction, deposits and soft tissue residues were removed from tooth surfaces by using a rubber cup and pumice slurry. The teeth were kept in an aqueous buffered solution of formaldehyde (Yekta Chem Co, Tehran, Iran) for two hours in order to decontaminate and then stored in normal saline at room temperature for three months before the laboratory procedures.

The roots of the teeth were sectioned 2 mm below the cemento-enamel junction (CEJ) with a low-speed water-cooled diamond disk (D&Z Diamant GmbH, Lemgo, Germany) and then the crowns were embedded in auto-polymerizing acrylic resin (Simplex Rapid, Kem Dent, Wiltshire, UK) with the buccal surface facing up and extending 2 mm above the resin surface. The enamel buccal surface was completely ground under water cooling to expose a flat dentinal surface using a straight fissure diamond bur (D&Z, Lemgo, Germany) placed parallel to the buccal surface. As the depth of dentin is a crucial factor affecting the dentin bond strength values and also for standardization, 1.5-mm depth holes were drilled in the middle of the buccal surface using a round diamond bur (D&Z) before grinding the dentinal surface flat. The cutting procedure was carried out at high speed under water cooling; the bur was held perpendicular to the surface and to ensure consistency of preparations the high-speed handpiece was attached to a modified surveyor. Subsequently, the dentin surface was wet-ground with 320-grit and polished with 600-grit silicon carbide sandpapers (Buehler Ltd, Lake Bluff, IL, USA), each for 30 seconds until no enamel remained to produce a uniform surface and standardized smear layer. Afterwards, all the samples were examined under a laboratory magnification lens to ensure that no enamel remained except at the periphery and/or exposed pulp tissue.

Prior to dentin surface treatment, an adhesive tape with a punch hole of 3.5 mm in diameter was applied on the flat dentin surface; then the samples were thoroughly washed and gently air-dried to remove excess water. Then the samples were randomly divided into five groups (n=15) according to the surface treatment performed: (1) Acid etching; (2) Laser etching; (3) Laser etching followed by acid etching, (4) Acid etching plus laser etching and (5) No acid etching and no laser etching (control group). In groups 1, 3 and 4, the dentin surfaces were etched by 37.5% phosphoric acid gel (Kerr Gel Etchant; Kerr Co., Orange, CA, USA). The acid was applied for 15 seconds, rinsed with air/water spray for 15 seconds and then blotted to remove excess water using a cotton pellet. In groups 2, 3 and 4, the dentin surfaces were conditioned by an Er:YAG laser device (Kavo Key Laser 3+, Kaltenbach & Voigt GmbH, Biberach, Germany) with a wavelength of 2.94 μm. The laser energy parameters were 140 mJ and 25 Hz with a pulse duration of 200 µs under air/water spray. The laser beam spot size was 2 mm and was moved in a sweeping fashion by hand over an area 3 mm in diameter by 2060 handpiece of the machine. The dentin surfaces were lased for 10 seconds in the non-contact mode perpendicular to the flat specimen surface with a 12-mm fixed distance from the laser tip. To ensure consistent energy density, spot size, distance, and handpiece angle, the laser handpiece was attached to a modified surveyor.

The samples in the first group were only acid-etched; the second group was only lased; the third group was lased and etched, the fourth group was treated in reverse order compared to the previous group; and the fifth group as the control group without acid etching and laser etching. Two consecutive coats of an etch-and-rise adhesive system (Optibond FL, Kerr Co) were immediately applied continuously to the etched dentinal surface for 20 seconds with gentle agitation using a fully saturated applicator. The surface was air-dried gently for 5 seconds to evaporate bonding solvents and then light-cured for 10 seconds using a light-emitting diode (LED) light-curing unit (Coltolux LED, Coltene/Whaleden Inc., OH, USA) with a light intensity of 800 mW/cm^2^, following the manufacturer’s instructions.

A transparent plastic tube with an inner diameter of 2 mm and a height of 2 mm was filled with a microhybrid resin composite (Point 4, Kerr Co, A3 Body Shade) in a one-layer incremental technique and attached perpendicularly to the prepared dentin surfaces. Subsequently, any excess resin composite was carefully removed from the periphery of the tubes with a sharp surgical blade. The tube was exposed to the curing light for 20 seconds vertically and for 40 seconds circumferentially (20 seconds from each side) to ensure complete polymerization. For all the specimens, the curing tip was placed as closely as possible to the composite resin surfasce. After the composite buildup, the plastic tube was carefully removed with a scalpel blade. The samples were kept moist to avoid drying and cracking during the laboratory procedures. All the samples were stored in distilled water at 37°C and 100% humidity for two weeks and then thermocycled for 1500 cycles at 5-55°C with a dwell time of 30 seconds in each bath.

The shear bond test was performed using a Universal Testing Machine (Zwick GmbH & Co, Ulm, Germany) at a crosshead speed of 1 mm/min in compression mode using a blunt knife-edged apparatus parallel to the adhesive interface between the adhesive and dentin. The maximum load to failure was recorded for each specimen and the shear bond strength was calculated in MPa, which is derived by dividing the force applied (the peak load) (in Newton) at the time of fracture by the bonded area (3.14 mm^2^). Data was statistically analyzed using parametric (one-way ANOVA) and post hoc Tukey multiple comparison tests) and nonparametric (Kruskal–Wallis and Mann–Whitney tests) at a significance level of 5%.

## Results

Means and standard deviations of the shear bond strengths of the tested treatment groups are shown in Table 1. Data analysis using parametric and nonparametric tests showed that statistically significant levels were the same by the two methods. Analysis of variance revealed statistically significant differences among the tested groups (P=0.001).

**Table 1:** Shear bond strengths (in MPa) of composite resin bonded to dentin, with no significant differences between groups with the same superscript letter

Groups	Mean ± SD	Min-Max
Acid etching^a^	20.1 ± 1.8	17.0–23.4
Laser etching^b^	14.1 ± 3.4	6.4–18.7
Laser etching + acid etching^b^	15.6 ± 3.5	9.9–20.8
Acid etching + laser etching^a^	21.5 ± 5.1	16.1–33.6
Control	8.1 ± 2.1	3.5–10.0

Statistical analysis between groups showed that the mean shear bond strength for acid-etched (1) and for acid+laser (2) groups were significantly higher than those in the laser+acid (3) (P≤0.004), laser-etched (4) (P=0.001) and control (5) (P=0.001) groups. Also, laser+acid (3) and laser-etched (4) specimens demonstrated higher mean shear bond strength values compared to the control specimens (5) (P=0.001). However, there were no significant differences in shear bond strengths between acid-etched and acid+laser groups (P≥0.05) and between laser+acid and laser groups (P≥0.05). Control group samples presented significantly lower shear bond strength values compared to other groups (P≤0.05).

## Discussion

The bond strength of adhesive systems is one of the major factors to be considered in the placement of composite resins.^22^ Adhesion of restorative materials to dental substrates is a desirable property because it is closely related to the prevention of material dislodgement and marginal leakage.^23^ An effective adhesion to tooth structure is of paramount importance to withstand the stresses resulting from polymerization shrinkage, thereby warranting retention and marginal integrity of restorations.^22^ Despite advances in the chemistry of adhesive systems, dentin remains a challenging substrate for bonding due to its heterogeneity.^24,25^ The bonding mechanism of composite resin to acid-etched dentin is well known and understood to be micromechanical.^26,27^ The formation of a hybrid layer and resin tags is essential to the establishment of a strong bond at the dentin level^12^ and may be achieved by complete dissolution of the smear layer and demineralization of intertubular and peritubular dentin by means of acid etching, resulting in an exposed collagen matrix which is infiltrated by resin that polymerizes in situ.^18,27^

Little is known about the adhesion of resin to laser-irradiated dentin, but it appears that the formation of an interdiffusion zone, which is the basis for dentin hybridization in acid-etched dentin, is unlikely. Instead, laser-irradiated dentin probably acquires its bond strength solely from the penetration of resin tags into dentinal tubules. Resin tag formation accounts for only a fraction of the bond strength in normal hybridized dentin.^28^ Cavity pretreatment with Er:YAG laser etching has been proposed as an alternative to acid etching of enamel and dentin. Some researchers have explored the use of lasers to modify the surfaces of teeth intentionally and improve bonding of restorations.^10,13,26^

The results of the current study clearly demonstrated that acid etching pretreatment alone is more effective than Er:YAG laser etching alone, and acid etching is able to enhance shear bond strength in dentin. Consistent with these results, Dunn et al^8^ reported that only the acid-etched specimens had significantly higher shear bond strengths, acid-etching was better than laser-etching, and laser-etching was better than not etching. In addition, Torres et al^16^ concluded that irradiation of primary dentin with the Er:YAG laser decreased the shear bond strength of total-etch and self-etching adhesive systems. Martínez-Insua et al^29^ confirmed that enamel and dentin surfaces conditioned by Er:YAG laser show extensive subsurface fissuring which is unfavorable to adhesion. Ceballos et al^11^ proposed that acid-etching alone yields shear bond strength values that are significantly higher than those achieved with laser ablation alone, or in combination with acid-etching. The laser-etching of dentin fuses collagen fibrils together, resulting in a lack of interfibrillar space, restricting resin diffusion into the subsurface intertubular dentin; therefore, it is not an alternative bonding strategy to conventional acid etching.

Although the shear bond strength of the acid+laser-etched group was a little higher than that of the acid-etched group, there were no significant differences. It is likely that laser etching after acid etching is not able to eliminate or improve the porosities created on acid-etched dentin. Also, this might be due to the honeycomb-like appearance produced by the application of acid etching and the additional scaly, rough zones and without thermal damage produced by Er:YAG laser pretreatment.^1^

The results of the present study did not show any significant difference between specimens etched with phosphoric acid followed by Er:YAG laser etching and Er:YAG laser alone. Torres postulated that acid-etching and water-rinsing steps appeared to have eliminated the surface laser-modified layer.^16^ However, acid etching after laser irritation is not able to eliminate the laser-modified layer completely.^30^ Moreover, the results indicated that acid-etched group had improved bond strengths compared with laser+acid-etched group. Acid resistance of the teeth increased after laser treatment;^8,15,31^ consequently, acid-etching might not totally expose the collagen matrix, especially in the peritubular region,^32^ and the adhesion to composite resin can be reduced by the lack of resin infiltration into the demineralized dentin.^33^

The present study revealed that shear bond strength of acid-etched dentin was significantly higher than that of laser-etched group alone. Consistent with the results of this study, previous reports have explained the low bond strength obtained in Er:YAG laser-etched dentin as a consequence of physiochemical changes caused by laser energy in the tissues.^8,11,15,16^ In contrast, some studies have shown the higher bond strength to laser-etched dentin than to acid-etched dentin; therefore, Er:YAG laser etching might eliminate the need for acid-etching dentin as a pretreatment for composite resin bonding.^34,35^

Current adhesive systems were originally developed to act on the tooth substrate prepared by conventional techniques; however, to create a more suitable surface when using the laser, adhesive systems should be improved or adherent surfaces should be modified.^1^ Shear strength test has been chosen instead of a microtensile bond test for the following reason: It is perhaps more clinically applicable because resistance to shear stresses is thought to be important in retaining restorations that have been bonded to tooth surfaces.^36^ Also it is more reproducible because crack initiation is more localized, and the interfacial crack propagates in a single direction, which is the same as the shearing test direction. For microtensile tests, cracks can initiate at multiple locations, which induces some discrepancies in the test results.^37^ Therefore, in the present study, a conventional shear bond test was selected as the parameter to measure how adhesive systems bond to dentin.

The results showed that acid etching of dentin prior to Er:YAG laser etching significantly improved the shear bond strength in comparison to acid etching followed by laser etching. Ceballos et al^11^ proposed that the laser etching of dentin fused collagen fibrils together; hence, it is probable that the phosphoric acid application is not able to improve laser-irradiated dentin surface and to create a rough microretentive pattern. Kataumi et al believed when laser-etched dentin is not separately acid-etched, collagen is not exposed and no hybrid layers can form.^32^

In the present study, specimens etched with Er:YAG laser followed by phosphoric acid etching showed significantly higher bond strength compared to specimens that were conditioned with Er:YAG laser alone. Therefore, acid etching after Er:YAG laser etching is necessary for improving adhesion, because of the enhanced bond strength values obtained when the two procedures are combined.^20,21^ In contrast to acids, which expose a microporous demineralized collagen fibril network that can be hybridized using conventional resin-based adhesives,^38^ the Er:YAG laser acts on dentin by thermo-mechanical ablation, vaporizing its water contents, which causes its rapid expansion followed by micro-explosions.^39,40^ Additionally, irradiation of dentin with Er:YAG laser includes the formation of a microscopically rough substrate surface without demineralization, open dentinal tubules without smear layer production, and dentinal surface sterilization.^11,37^ This ablation process leaves no hydroxyapatite-depleted collagen on the surface^39^ and exhibits less regular and less homogenous aspect with subsurface fissures resulting from heat generated during irradiation, which might also be adverse factors for adhesion.^6^

## Conclusion

Within the limitations of this laboratory study, it may be concluded that:

1. Phosphoric acid etching is still an effective dentin pretreatment technique for composite resin restorations.

2. Acid etching followed by Er:YAG laser pretreatment did not enhance the adhesion of composite resin to dentin surface compared with acid-etching alone.

3. Pretreatment of dentin surface with Er:YAG laser prior to acid etching did not improve shear bond strength compared with laser-etching alone.

Therefore, when the cavity is prepared by bur, it is not necessary to etch the dentin surface by Er:YAG laser following acid etching and acid etching after laser etching.

## Acknowledgements

This study was supported by a grant of Research Vice Chancellor of Rafsanjan University of Medical Sciences (Grant No: 90.20.552). The research was funded by Rafsanjan University of Medical Sciences. The authors express their gratitude to Dr. S Rahimi for helping in laboratory procedures. The authors declare that they have no conflict of interests in this research.
